# Field Translocation of Mountain Pine Beetles Suggests Phoretic Mite Communities Are Locally Adapted, and Mite Populations Respond Variably to Climate Warming

**DOI:** 10.3390/insects12020131

**Published:** 2021-02-02

**Authors:** Sneha Vissa, David N. Soderberg, Richard W. Hofstetter

**Affiliations:** 1 School of Forestry, Northern Arizona University, 200 E Pine Knoll, Flagstaff, AZ 86011, USA; Rich.Hofstetter@nau.edu; 2 Wildland Resources Department, Utah State University, 5230 Old Main Hill, Logan, UT 84321, USA; davidsoderberg@gmail.com

**Keywords:** *Dendroctonus ponderosae*, mite communities, Acari, phoresy, climate warming, field reciprocal translocation, local adaptation

## Abstract

**Simple Summary:**

Climate warming has significant effects on forest insect populations, particularly bark beetles, which cause millions of hectares of forest tree damage. Bark beetles live alongside a diverse host of other organisms which affect the success of beetle attacks on trees and are also affected by climate changes. Here, we explore climate effects on symbiotic mite communities associated with the mountain pine beetle (*Dendroctonus ponderosae*). We show that warming causes significant shifts in the abundance of mites. These effects were dependent on source population, suggesting mite populations are adapted to their local climates. Understanding beetle–mite patterns is important because mites can directly affect beetle reproduction by feeding on eggs, or indirectly affect beetle health by introducing fungi. Our results provide foundational information for understanding how climate change will affect beetle–mite associations; and serve to help determine how these shifting associations will affect the success of bark beetles in forest ecosystems.

**Abstract:**

Temperature is a key determining factor in the population dynamics of forest insects and their associated biota. Bark beetles, often considered key agents of change in forest ecosystems, are particularly affected by warming in their environment. Beetles associate with various phoretic mite species that have direct/indirect effects on beetle fitness and population dynamics, although there is limited knowledge of how temperature affects these communities. Here, we use a field reciprocal translocation experiment with the addition of a novel “warming” environment to represent future changes in local environment in two populations of a keystone bark beetle species (*Dendroctonus ponderosae*). We hypothesize that mite community abundances as carried by bark beetles are significantly altered when not in their native environments and when subjected to climate warming. We use multivariate generalized linear models based on species abundance data to show that mite community compositions significantly differ across different field climates; and that these patterns diverge between source populations, indicating local adaptation. Our study offers foundational information on the general effects of simulated climate-warming on the compositional shifts of common and abundant biotic associates of mountain pine beetles and may be used as a model system for other important insect–mite systems.

## 1. Introduction

Temperature is an important factor in insect ecology [1,2,3,4]. Across North America, changes in seasonality driven by increases in temperature have resulted in longer growing seasons, rapid phenotypic adaptation, and range expansion of a variety of arthropod species [5,6,7]. Adaptation and range expansion on a landscape scale are evident from bark beetle (Coleoptera: Scolytinae) attacks in forest ecosystems across North America [7,8,9,10]. Native bark beetle species have coevolved with conifer forests and are key agents of change in these systems [11,12]. Critical events for population turnover, such as adult emergence, flight patterns, aggregation, and reproduction of these beetles, are highly dependent on temperature [13,14,15]. Warming, in particular, has allowed high-elevation bark beetle species, such as the mountain pine beetle (*Dendroctonus ponderosae* Hopkins), to expand their ranges [16,17,18,19,20], causing millions of hectares of forest tree damage [21,22,23,24].

*Dendroctonus ponderosae*, whose range spans most of western North America, is considered a keystone species with the potential to influence forests on a landscape level [19,25]. These beetles exhibit resilience to short-term changes in climate regimes [26], distinct genetic differences across populations [27], and trait phenotypic plasticity [9]. They are also directly associated with an array of symbiotic organisms (symbiota), forming a biodiverse community comprising multiple interactions that directly and indirectly influence their (beetle) performance [28,29,30]. Temperature is a determining factor for the presence/absence and fitness of these symbiota as well [16,31,32]. Increase in temperature not only impacts beetle development directly [33,34], but indirectly affects beetle performance through influences on its symbiota [35].

Phoretic mites are a particularly abundant associate of *D. ponderosae* [29,36,37], forming a spectrum of symbiotic interactions (i.e., acting as mutualists, antagonists, and/or commensalists) [30,38]. Mites latch on to adult beetles prior to emergence from the tree and are carried into new tree environments where they are deposited into new beetle galleries [39,40,41]. Mites reproduce in these galleries, sharing and competing for niche space within the bark beetle system [30,36]. Beetle fitness may be positively and/or and negatively correlated (depending on the beetle species) with the abundance of mites and the presence of specific mite species, particularly those species that parasitize beetle eggs or have the potential to alter the fungal environment [42,43,44,45]. However, unlike other biological associates, particularly the mutualistic fungi [46,47], there is limited information on climate- and temperature-driven patterns in phoretic mites [31,48]. Similar to how mutualistic fungi of *D. ponderosae* (and the beetles themselves) exhibit variability in their fitness and mutualistic associations across different populations [6,10,35], the overall mite community composition of these beetles also varies; with these differences largely driven by species replacement or, in some cases, species elimination [37]. Differences in climatic factors, particularly temperature, can often explain shifts in species composition but this remains untested on a population level for phoretic mite associates of *D. ponderosae*.

In this study, we explore shifts in beetle–mite communities driven by climate warming by using a reciprocal field translocation experiment to identify whether direct changes in the local environment, specifically temperature, affect the mite composition on emerging adult *D. ponderosae* in the southwest United States; and whether mite abundance and composition are determined by local adaptation to native environments. We additionally measure effects of warming temperatures on mite communities with a novel field translocation treatment outside the beetle’s historic distribution range to simulate “climate warming” in its native range. *Dendroctonus ponderosae* populations from Utah and Arizona, though distinctly different populations [9,26], have nested mite communities [37]; therefore, we asked whether shifts in mite community composition could be explained by differences in their local environment and climate warming. We hypothesized that the mite community compositions on *D. ponderosae* (based on the presence and absence of species and their abundances) are significantly affected by changes in their local climate. We specifically predicted that: (1) warming causes a significant shift in the abundance of mite species; (2) mite communities are locally adapted to their native beetle environments, and therefore the observed patterns of shifts in mite communities across different field environments will vary between source populations.

## 2. Materials and Methods

**Figure 1:**
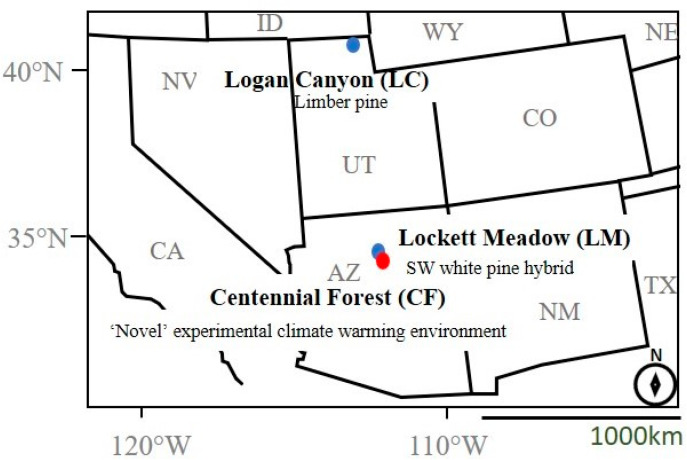
Field sites for translocation experiment. Blue circles on Logan Canyon, Utah (LC) and Lockett Meadow, Arizona (LM) indicate the origin of the two beetle populations in this study. Centennial Forest, Arizona (CF), shown as a red circle, serves as the third “novel” experimental climate-warming environment for both populations.

We used two populations of *D. ponderosae* from Utah (UT) and Arizona (AZ) within the beetle’s known range in the southwestern United States for our field translocation experiment. The populations are latitudinally separated, with Arizona being the more southern population of the two (Figure 1; Table 1).

### 2.1. Parent Beetle Collection and Host Tree Environment

We identified two high-elevation five-needle white pine trees (*Pinus flexilis* E. James in UT and *Pinus strobiformis* Englm hybrids in AZ [49]) infested with *D. ponderosae* in fall 2016. Beetle infestations were confirmed using visual characteristics [50]. In spring 2017, we cut the infested *P. strobiformis* hybrid trees and stored them upright in emergence containers in the Northern Arizona Forest Entomology Lab. Similarly, we cut and stored infested *P. flexilis* at the Rocky Mountain Research Station (RMRS) Laboratory in Logan, Utah in spring 2017 prior to adult beetle emergence.

*P. flexilis* and *P. strobiformis* are closely related high-elevation five-needle white pine species with similar morphological and physiological characteristics [51,52]; and were even considered subspecies of *P. flexilis* until recently [51]. Active gene flow between the two species indicates successful reproductive hybrids (such as the *P. strobiformis* hybrids found in Northern Arizona) [49]. There is also substantial evidence of local adaptation to heterogeneous climatic conditions within populations of these tree species, largely explaining the divergence between the two species [49]. Differences between trees are therefore more attributable to differences in climatic conditions. Differences in *D. ponderosae* host preferences across its geographic distribution are also credited to differences in local climate [15]. We consequently rationalize that differences in the internal host tree environment, regardless of whether they are *P. flexilis* or *P. flexilis x P. strobiformis* hybrids, are most likely to be influenced by differences in local climate and introduced beetles into host trees harvested from their native environments for the translocation experiment.

### 2.2. Translocation Preparation

**Figure 2:**
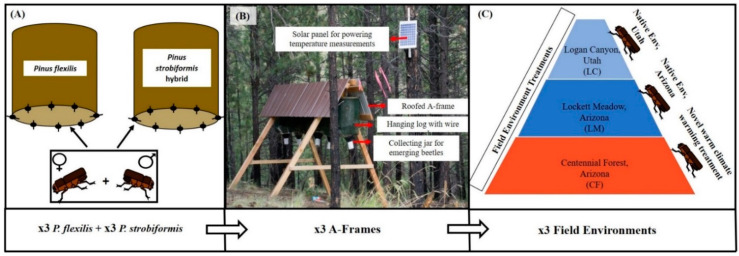
Methods for experimental field translocation experiment, providing details on beetle infestation in logs (**A**); A-frame structures used to hang logs (**B**); and field treatments, where red signifies warmer temperatures and blue tones signify cooler field temperatures, on average, based on maximums and minimums as listed in Table 1 (**C**).

Upon beetle emergence in August 2016, we collected and sexed mountain pine beetles from each population based on acoustic and morphological sex characteristics [53,54]. We collected beetles daily and stored live beetles in Petri dishes lined with moistened filter paper at 4 °C for no longer than 10 days. For each beetle population (AZ and UT), we harvested three healthy, un-attacked *P. flexilis* and *P. strobiformis* hybrid trees from the original beetle collection locations (LC and LM; Figure 1), approximately 38 cm diameter at breast height, and cut them into logs measuring ~46 cm in length. To seal in moisture, we coated the exposed cut-ends of the logs with paraffin wax (Gulf Wax ^®^). We introduced male–female beetle pairs into logs of their natal host tree environment, as described below (Figure 2A).

We prepared logs by drilling ten to twelve 5-mm holes, ~6 cm apart, into the phloem at the anatomical bottom end of the log. These holes served as entry points for mating beetle pairs. Females are the first to arrive at a tree to make entry points, while males follow in response to female pheromones [50]. Therefore, we introduced females into the prepared holes first, allowing them to burrow in before introducing male beetles behind them and sealing the holes using a fine plastic mesh.

### 2.3. Reciprocal Translocation and Field Environmental Profiles

Of the 54 total logs, we retained nine logs from each population in their native field environments. We translocated another nine logs from each population, by swapping between LC and LM. We placed the remaining nine logs from each population in a third “novel” environment just outside the beetle’s historical range, but still within its potential range, located at Centennial Forest (CF), Northern Arizona at an elevation lower than that of LM, in ponderosa pine (*Pinus ponderosa*) forest habitat. This location served as a proxy for “climate warming” in both populations (Figure 2C; Table 1). While *D. ponderosae* have not historically occurred in Northern Arizona ponderosa pine habitat, this serves as a proxy for future warming in their native higher elevation habitats, where warming may cause their native host trees to become more susceptible to beetle attack. *Dendroctonus ponderosae* are also capable of attacking ponderosa pine trees at other geographic locations [55,56].

At each field site, we used three large A-frame structures to hang the logs and protect them from harsh weather. We randomized and hung logs such that there were an equal number of Utah and Arizona logs under each A-frame (Figure 2B). We placed the logs at each of the three field sites (LC: 30th July, 2016; LM: 10th August, 2016; and CF: 11th August, 2016) for ~1 year (duration of beetle development) until the emergence of the next generation of beetles. All bolts were protected from external disturbance by a plastic mesh covering around individual logs, equipped with an opaque collection jar at the bottom for emergence (Figure 2B).

To determine the overall temperature profiles of the three field sites, we collected air and internal log temperature using CR1000 measurement and data loggers (Campbell Scientific, Logan, UT, USA) which recorded data hourly for the duration of the experiment, specific details of which are available in a previously published study [26]. We used these data to calculate daily maximum and minimum temperatures for the beetle log environment in all three field sites to make inferences on environmental temperature effects. The native environment of UT beetles at LC was, on average, the coldest site with the lowest average minimum temperature (Table 1). LM, the native environment of AZ beetles, had warmer winter temperatures on average than LC. The novel warm environment (CF) had the warmest temperatures on average with the highest average maximum temperature, although minimum temperatures were similar to those of LM, and served as the novel warming environment for both populations (Table 1). Internal log temperatures did not vary within sites [26].

Detailed beetle emergence patterns (synchrony, development rate, genetic differences in populations) have already been published [26]. Here, we present successful beetle emergence per site estimated based on the number of beetles per site and the number of successful galleries (Figure S1) per site to provide information about mite abundances relative to beetle emergence across sites and populations.

### 2.4. Mite Collection and Identification

In July–August 2017, approximately one year after the experimental setup, we collected emerging *D. ponderosae* from the reciprocal translocation study logs. We collected emerging beetles every 4 days during non-peak emergence and every 2 days during peak emergence. We stored beetles in individual gel capsules in the freezer for mite collections.

From the total number of emerged beetles, we sampled a subset of 100–107 beetles for mites from each population and location. Note that the remaining emerged beetles from these logs were used in a study identifying local climate effects on beetle physiology and fitness that is already published [26]. We counted and identified each mite using a dissecting microscope (for more information on the specific anatomical attachment of these mites, see Figure S1). We transferred all mites onto glass slide mounts with lactic acid medium. We used lactic acid to degrade mite surface proteins and reveal internal structures for species identification. We identified mites to the highest possible taxonomic resolution and were assisted by numerous mite taxonomists (see acknowledgements) and other identification sources [57,58]. We stored voucher specimens at the Forest Entomology Lab at Northern Arizona University’s mite collections and imaged voucher specimens at the Museum of Northern Arizona for identification purposes.

### 2.5. Data Analysis

We used a generalized linear model (GLM) in the *MASS* package [59] to analyze beetle emergence data, and a negative binomial generalized linear model (GLM) with two predictor variables for multi-variate data in the *mvabund* package for RStudio [60] to assess differences in mite species abundances across populations and field sites. The principal model fitting function, *manyglm*, allowed us to obtain population-level global estimates for differences in community composition through multiple species testing while treating beetle population and field sites as interacting effects (Avg_abundance_ = Population × Field Treatment; where “Population” and “Field Treatment” are explanatory variables). The negative binomial distribution best fit the data, accounting for the numerous zeros and overdispersion of variances. Model assumptions were met and confirmed by QQ-plot and Dunn-Smyth residuals generated by the plot function in the *mvabund* package [60]. This method also provided individually fitted GLMs for each mite species within and across treatments and populations. To account for correlation in testing, we used one-thousand bootstrap iterations via “pit.trap” resampling—a bootstrap procedure for regression models with discrete, multivariate responses [61]. We regarded weak correlations as insignificant in the case of very rare species.

We used an analysis of variance (ANOVA) to determine site level differences in beetle emergence data, and Tukey’s HSD test for pairwise differences in beetle emergence. For mite pairwise comparisons, we determined significant differences using the ANOVA function (“anova.manyglm”) built into the *mvabund* package (ver. 4.1.6, https://CRAN.R-project.org/package=mvabund). This also provides pairwise comparisons using a free stepdown resampling procedure. We conducted all analyses in R ver. 3.6.2 (R Core Team, 2019, <CRAN MIRROR>/bin/windows/base/release.htm), using average emergence of beetles per week and raw mite abundance data. Mite abundances were pooled across all logs within a field environment as the internal phloem temperature of logs did not vary within sites [26]. We used the total number of successful galleries per site (Figure S1) and the total number of beetles emerged to calculate a metric for beetle emergence success.

## 3. Results

### 3.1. Beetle Emergence Patterns

**Figure 3:**
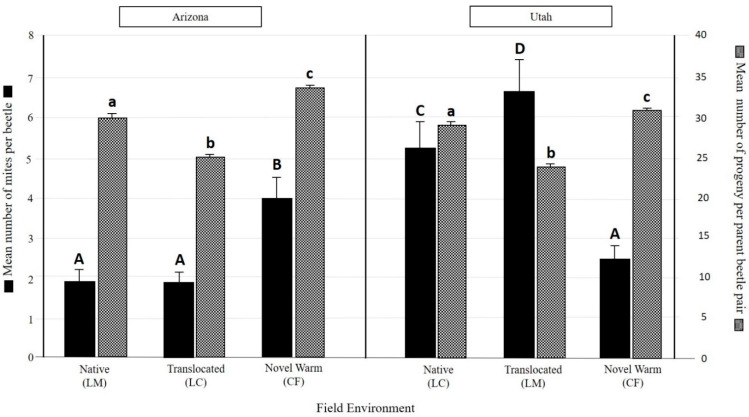
Beetle emergence success shown as the mean number of successful progeny per parent beetle pair (checkered bars), and mean number of mites per beetle (black solid bars) for three field climate treatments. LC = Logan Canyon (native environment for UT beetles and mites); LM = Lockett Meadow (native environment for AZ beetles and mites); and CF = Centennial Forest, a novel environmental treatment simulating “climate warming” in the field. Error bars represent standard error. Upper case letters show significant differences in mean number of mites per beetle across treatments and between populations. Lower case letters similarly show significant differences in number of progeny per parent beetle pair.

Beetles performed best at the novel warm site (CF) for both populations, as reflected in the higher number of progeny per successful parent beetle pair in the novel warming climate, and there was no significant difference between the average number of beetle progeny between populations (Figure 3). Utah beetles had fewer successful progeny per parent beetle pair when translocated to LM, compared to their native environment at LC. For the AZ population, there were fewer beetle progeny per pair at LC translocation environment (Figure 3) which was, on average, cooler than its native environment at LM (Table 2).

### 3.2. Overall Mite Community Differences

Five mite taxa were found across both beetle populations; four of which were identified to the species level (Table 2). Of these, one species, *Tarsonemus endophloeus* Lindq, only occurred with UT beetles (Table 2) and were rare (<0.1 mites per beetle). Five mite taxa were observed on UT beetles, and four mite taxa on AZ beetles (Table 2). On average, more phoretic mites were associated per beetle in the UT population than the AZ population in all environments except the novel warm environment (Figure 3). Information on the specific morphology and attachment of mites on beetles can be found in the Supplementary Materials (Figure S2).

Using the total number of beetles emerged from each site and the number of successful galleries (i.e., the number of successful parent beetle pairs from the initial inoculation), we calculated the number of mites found in each successful parent beetle gallery as an estimate of mite success at each field treatment. We found that AZ beetles in the novel warm climate treatment had twice the number of mites per beetle gallery than their native environment (Table 2). Utah populations, on the other hand, had more than twice the number of mites per gallery in their native environment (LC), compared to the novel warming environment (CF) where they had significantly fewer mites per beetle gallery (Table 2).

Mite community composition (based on a cumulative global estimate from individual species GLMs) significantly differed between AZ and UT populations (Dev = 45.76, df = 610; pr (>Dev) = 0.001) and among three field environments (Dev = 34.07; df = 608; pr (>Dev) = 0.001). Interaction of population origin and field environment also had a significant effect on mite community (Dev = 65.94; df = 606; pr (>Dev) = 0.001). More phoretic mites were associated per beetle in the UT population than in the AZ population in all environments except the novel warm environment (Figure 3). Mite abundances per beetle were significantly greater in the novel warm environment (CF) than both the native environment (LM) and the coolest field environment (LC) for AZ beetles (Figure 3).

Average overall mite abundances per beetle were significantly higher in the native environment (LC) and at the translocated environment in LM than the novel warm environment (CF) for UT populations (Figure 3).

### 3.3. Individual Species Differences within and Across Populations

**Figure 4:**
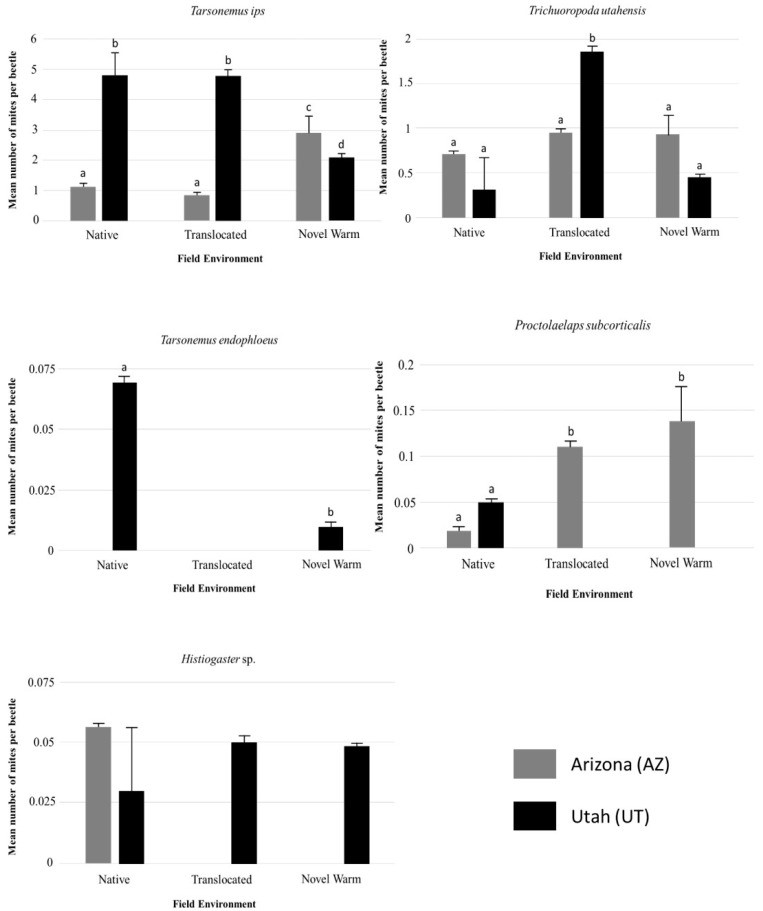
Mean number of mites per beetle for individual mite species in each field environment for Arizona (AZ) and Utah (UT) populations. LC = Logan Canyon (the coldest environment on average and native environment for UT population); LM = Lockett Meadow (native environment for AZ population); and CF = Centennial Forest (novel experimental climate warming environment for both populations). Bars represent standard error; and letters indicate significant pairwise differences within field environments and between populations.

*Tarsonemus ips* Lindq was the most abundant mite in both populations, with 1.13 (±0.2) mites per beetle, with an estimated 34 mites per successful beetle gallery in the native AZ population (Table S1); and 4.8 (±0.63) mites per beetle and with an estimated 139 mites per successful beetle gallery in the native UT population. The mean number of mites per beetle differed significantly between populations (Table 3), and across treatments within populations (Table 3; Figure 4). The abundance of these mites increased significantly in the novel climate warming environment (CF) for Arizona populations with nearly double the mites per successful gallery; however, the number of mites reduced by more than two-fold at the novel warming environment for the UT population (Table S1; Figure 4).

*Tarsonemus endophloeus* Lindq was the rarest mite species, with 0.07 (±0.04) per beetle and between 0–1 mites per successful beetle gallery where they occurred (Figure 4; Table S1). *T. endophloeus* was only found in the native environment for UT beetles (Figure 4; Table S1). The abundance of these mites showed slight differences between the two populations (Table 3) and across treatments (Table 3).

*Trichouropoda utahensis* Wis & Hirsch was the second most abundant mite species on both beetle populations (although they were more abundant in AZ than in UT), with 0.71 (±0.17) mites per beetle with an estimated 21 mites per successful gallery in the native environment for AZ beetles; and 0.31 (±0.08) mites per beetle and an estimated 9 mites per successful gallery in the native environment for UT beetles (Figure 4; Table S1). Phoretic *T. utahensis* associated with Utah beetles differed significantly across environments (Table 3; Figure 4) but did not differ significantly between environments for Arizona beetles (Table 3). Their abundances significantly differed within the same environment between AZ and UT populations (Table 3; Figure 4).

*Proctolaelaps subcorticalis* Berlese abundances varied significantly between beetle populations (Figure 4;Table 3) and across field environments within beetle populations, although this was less significant (Figure 4; Table 3). There was no interaction between population and environment (Table 3). Abundances of this mite species were extremely low across both populations, with 0.02 (±0.01) mite per beetle with an estimated 0–1 mites per successful beetle gallery on AZ beetles in their native environment; and 0.05 (±0.04) mites per beetle and an estimated 2 mites per beetle gallery in the native environment for UT beetles.

*Histiogaster* sp. were also very low in abundance, and only found in the native environment for the AZ population. A total of 0.06 (±0.03) mites per beetle, with an estimated two mites per beetle gallery on the AZ beetles in their native environment; and 0.03 (±0.08) mites per beetle and an estimated one mite per successful gallery were found at the native environment of UT beetles (Figure 4; Table S1). The abundance of these mites did not vary significantly across field environments or between populations within a field environment.

## 4. Discussion

Predicting community responses to climate change is challenging, as multiple factors influence species responses to temperature [62,63,64]. These patterns are dynamic and often inconsistent between species and across populations [65,66,67]. Even with controlled laboratory experiments that tease apart specific trait-based responses to environmental changes, patterns are not always clear and may not be applicable in a field setting [66,67,68,69]. Here, we used a field reciprocal translocation experiment to address potential future changes in climate and how climate warming may affect mite communities associated with a keystone bark beetle species [70,71] in western North America—the mountain pine beetle, *Dendroctonus ponderosae*. Patterns in mite abundance and composition were not consistent across beetle populations and environment treatments.

### 4.1. Temperature, Beetle Emergence, and Mite Abundance

Mite taxa identified in our study were consistent with those identified in other *D. ponderosae*–mite studies [29,36,72]. The overall total diversity of mites was low for both populations, although this may be a facet of manipulating and restricting the number of beetle pairs in each treatment [31], which may exclude low abundance and rare species that were not introduced during the experimental setup. Differences in the abundances of phoretic mites associated with the original parent beetles may also explain why UT beetles consistently had significantly more mites per beetle on average and a much higher number of mites per individual beetle gallery than AZ beetle populations. However, caution should be exerted in the interpretation of these mite success metrics. Although not clearly documented in this study, the frequency of mites may vary across the emergence period with more mites per beetle during peak emergence and fewer mites per beetle in non-peak emergence periods. Secondly, mite abundances may also vary with beetle reproductive success across different populations. Other factors, such as fungal presence and composition within trees, could also affect mite abundances on emerging beetles [31,48,73].

The environmental temperature profiles show that the native environments of both beetle populations were, on average, cooler than the novel warming environment. All three environments had varying temperature profiles which can directly affect mite development as mites are sensitive to fluctuations in temperature [31,48,74]. In general, our hypothesis was supported in that phoretic mite populations varied in response to warming and change in local climate within and across different populations. As predicted, beetle-associated mite communities differ significantly within and across beetle populations, across temperature regimes, and within temperature regimes between populations. Mite abundances generally appeared to increase with warming in the AZ populations but decreased with warming in UT populations, indicating that mites may have divergent responses to changes in local environment. Climate warming caused an observed increase in the abundance of *Tarsonemus* mites per beetle along with an increase in the number of beetles per gallery in AZ populations. Contrarily, we observed a decrease in mite abundances per beetle in the same climate warming environment for the UT population (although UT populations also exhibited increased beetle progeny per gallery in this environment). Similarly, abundances of *Trichouropoda* mites fluctuated with climate warming differently between the UT and AZ populations, despite high numbers of beetle progeny in the climate warming treatment for both populations. This suggests that, on a species level, mite populations have locally adapted responses to climate warming, and that the mite populations of AZ beetles may be better adapted to warming than their UT counterparts. This in turn may be explained by local adaptation to cooler climates on average in UT source populations; or by potential differences in fungal composition and growth within trees in response to changes in temperature regimes [35,75,76,77].

Few bark beetle–mite studies report the relationship between beetle emergence and relative mite abundance [31,78]. Mite abundances can increase with beetle emergence in *Ips grandicollis* bark beetles, suggesting that these mites and beetles exploit a mutually beneficial environment [78], although this is contrary to observed patterns in *D. ponderosae* populations [29,37]. While both beetle emergence and mite abundances per beetle increased with climate warming for AZ *D. ponderosae*, this pattern was inconsistent with the UT beetle population, where beetle emergence was favored by warming but mite abundances per beetle were not. This suggests that while warming in the local environment may be mutually beneficial for beetle–mite associations in some populations, they may be variably adapted in other populations of the same beetle species. Similarly, a field study testing the effect of temperature on mite communities associated with southern pine beetles (*Dendroctonus frontalis* Zimm.) showed an increase in temperature can cause a decrease in the abundance of *Tarsonemus* mites [73]. *Trichouropoda* mites, on the other hand, increased with increase in temperature [73]. These effects may also be driven by the associated fungal performance in specific temperature regimes as these beetles and most mite species are dependent upon fungi for resources within the tree [31,41,48,73]. Thus, *D. ponderosae* mite population patterns may be differentially adapted to environmental changes and driven by unknown underlying genetic and/or abiotic factors rather than a direct effect of temperature or beetle emergence alone (see “ecological implications” below).

As suggested by studies in the *D. frontalis* system [31,48], mites are sensitive to temperature, with some mites being better suited for warmer temperatures than others. *Dendroctonus ponderosae* and *D. frontalis* both attack multiple *Pinus* tree species [79], and carry mites with similar taxonomy, morphology, and function [24,30,48]; therefore, patterns in mite associations are comparable across beetle species. Lockett Meadow (LM), AZ, for example, seemed to be most favorable for *T. utahensis*, and the novel warm environment (Centennial Forest (CF), AZ) the least favorable; whereas *T. ips* was favored by the novel warm climate treatment. Arguably, these patterns compliment the environmental filtering hypothesis [76], which suggests shifts in species composition, particularly the loss of species (reflected in significantly reduced abundances), across populations is explained by differences in local environmental conditions and variation in the adaptation to these local environments. However, methods for parsing the outcome of biotic interactions from climate and temperature effects remain insufficient [80]. Given what we know of the fungal associates of these bark beetles and their responses to temperature, it is possible that relative performance of fungi within these logs may also be driving beetle–mite fitness and progeny abundances [29,31,69,81]. Further investigation is needed to confirm fungal effects on mite communities in *D. ponderosae* and other similar bark beetle systems in relation to climate change.

### 4.2. Ecological Implications

*Dendroctonus ponderosae* is a “mycangial beetle” (i.e., beetle possessing specialized structures for the transfer and inoculation of fungi) and relies on mutualistic fungi for nutrition and to potentially help neutralize tree defenses to overtake host trees [82,83,84]. This has consequences for niche exploitation, coexistence, and a causal chain of complex interactions dependent on the community of symbiotic species associated with the host beetle population [43,85,86]. *D. ponderosae* are associated with three known mutualistic blue-stain fungi, *Ophiostoma montium*, *Grosmannia clavigerum*, and *Leptographium longiclavatum* [87,88,89]; however, these fungi exhibit variability in their mutualistic associations with beetles across different populations [27,35], largely due to differences in fungal performance driven by warming [32,35,75,76,77]. Therefore, while warmer temperatures favor the performance of some fungi (e.g., *O. montium*), cooler temperatures serve as optimal conditions for others (e.g., *G. clavigera* or *L. longiclavatum*) [35,77]. Compared to the other common fungal associates, *G. clavigera* is the most nutritionally beneficial for *D. ponderosae* [82], but is not likely to occur in beetle populations that are in warmer climates (i.e., beetle populations at lower latitudes) where it is likely to lose its competitive advantage to *O. montium* [32,35,77].

Fungivorous (fungus-eating) and fungus-carrying mites, such as *Tarsonemus* sp., *Proctolaelaps* sp., and *Trichouropoda* sp., also form similar associations with blue-stain fungi [30,31,81,85,90,91]. These associations can subsequently alter the fungal environment within beetle galleries, leading to cascading effects on beetle–fungal associations and beetle fitness [30,38,41,43,92]. Climate-driven shifts in fungal performance can therefore influence mite–fungal associations and mite fitness, particularly for species that feed on and/or vector fungi. While beetles may be adapted to one of three different fungi, mites may only hold preference for one or two. Although *G. clavigera* is more nutritionally beneficial than either *O. montium* or *L. longiclavatum* for *D. ponderosae* beetles, Reboletti [93] found that *T. ips* associated with these beetles preferred to feed on and transfer *O. montium* rather than *G. clavigera*. This suggests the relative performance of these fungi within our study trees could drive phoretic mite abundance patterns. It is unknown whether *L. longiclavatum* is a suitable resource for *T. ips* or other mite species, but in other conifer-infesting bark beetle systems, *Leptographium* fungi are not often carried by phoretic mites [94].

Mites can directly and indirectly affect beetles either through predation of beetle eggs, nematodes, other mites, and through the introduction and spread of mutualistic or antagonistic fungi [41,44,80,95], thereby altering the fungal environment of beetle galleries. The introduction of *O. minus* fungi by *Tarsonemus* spp. in the galleries of *D. frontalis* beetles, for example, could create indirect negative feedbacks for *D. frontalis*, as it may outcompete the beetle’s mutualistic *O. ranaculosus* fungus [38,44,96]. Similarly, the introduction of *O. novo-ulmi* fungus (Dutch elm disease) by *Tarsonemus* and *Proctolaelaps* mite species negatively affects *Scolytus* spp. bark beetle fitness in *Ulmus* trees by rapidly killing the host tree [80,81,82,83,84,85,86,87,88,89]. Mites may also show preferences for specific host tree environments, although these preferences are not clearly defined [29,97]. Mite composition has been shown to vary between distinctly different host tree species, largely associating with beetles that colonize their preferred tree environment [29,97], although there is overlap in generalist mite species that can be found across multiple beetle/host tree environments.

Different symbiotic species also have variable strategies for niche exploitation and coexistence [86,98], and modify the beetle’s environment in different ways. This creates the potential for a series of eco-evolutionary feedbacks [98] driven by interactions across the spectrum of symbiosis [47]. It may be argued that the drivers of these interactions (the mite community of bark beetles) are innate, i.e., passed from parent beetle environment to the offspring’s environment, because of the strong phoretic association between mites and beetles [41]. The resulting feedback on beetle populations is therefore likely determined by the specific functional roles of the mites introduced into the beetle environment, the frequency of these mite functional groups (i.e., the abundance of predators, fungivores, etc.), and the presence of other organisms within the tree. Mites that alter the beetle–fungal environment within the tree are indirectly shaping the beetle environment by influencing fungal colonization patterns via the introduction of competing species [30,38,41,99]. Long-term positive and negative ecological feedbacks therefore depend on the phoretic species, their competitive advantage relative to other beetle associates, and by the environmental conditions that drive these interactions. The resulting causal chains of interactions in biotic communities can be important in determining selection for species and coevolution between species within an ecological community [82,98,100]. Beetle–fungal interactions are linked to such large-scale causal chain effects [86]; by the sheer frequency of association, it may be that mite interactions also hold larger ecological and coevolutionary consequences for forest bark beetle systems. The challenge that remains, however, is identifying the specific interaction pathways that determine these causal chains of events on an ecosystem level.

## 5. Conclusions

Differences in local environmental temperatures, particularly climate warming, can have a significant effect on the mite assemblages of *D. ponderosae* beetles. These effects differ based on the source population of beetles, suggesting that the community composition of phoretic mites are locally adapted to beetle populations, and therefore respond differently to the climate warming scenario. While assessing and separating the influence of environmental and genetic effects, along with biotic and abiotic factors, on long-term ecological and evolutionary processes remains a challenge; observational field experiments such as ours provide foundational information for predicting community shifts in common biotic associations of ecologically and economically relevant bark beetles in the face of anticipated warming in forest ecosystems.

## Figures and Tables

**Table 1:** Detailed information on source populations, translocation environments, and novel experimental warming environment for the reciprocal translocation experiment. Population and field environment codes as stated in this table are frequently used in explaining results.

Field Location	Relevance to Populations	Host Tree (*Pinus* sp.)	Latitude (N)	Longitude (W)	Elevation (m.)	Total Mean Temp (°C)	Average Min Temp (°C)	Average Max Temp (°C)
Logan Canyon (LC), UT	Native (UT)	*P. flexilis*	41.93	111.44	2204	4.7	−28.5	30.4
Lockett Meadow (LM), AZ	Native (AZ)	*P.strobiformis* hybrid	35.35	−111.62	2604	9.2	−14.2	28.6
Centennial Forest (CF), AZ	Novel Warming	(*P.ponderosa* habitat)	35.14	−111.71	2106	10.2	−14.2	32.8

**Table 2:** Mite collection information including total number of beetles examined for mites, percent mite occurrence per beetle, and estimated projected total number of mites per gallery in each population for all three field environments. The estimated mites per gallery is the number of mites associated with each successful parent beetle gallery per site.

Population	Field Environment	Total No. Beetles Examined	% Beetles with Mites	Estimated Mites per Gallery	List of Species Associated with Populations
Arizona	Native (LM)	107	59.8%	56.7	*Tarsonemus ips* Lindq.
					*Trichouropoda utahensis* Wis. & Hirsch.
	Translocated (LC)	100	66%	59.5	*Proctolaelaps subcorticalis* Berlese
					*Histiogaster* sp.
	Novel Warm (CF)	101	63.4%	131.3	
Utah	Native (LC)	101	72.3%	149.5	*Tarsonemus ips* Lindq.
					*Tarsonemus endophloeus* Lindq.
	Translocated (LM)	100	80%	167.9	*Trichouropoda utahensis* Wis. & Hirsch.
					*Proctolaelaps subcorticalis* Berlese
	Novel Warm (CF)	103	68%	57.5	*Histiogaster* sp.

**Table 3:** Analysis of variance tables for individual species generalized linear models (GLMs) by population (i.e., Arizona vs. Utah populations), and across field environment.

Variable	DF	Dev	Pr (>Dev)
*Tarsonemus ips* Lindq.			
Population	610	29.08	0.001 **
Field Environment	608	1.6	0.441
Population: Field Environment	606	32.35	0.001 **
*Trichouropoda utahensis* Wis. & Hirsch.			
Population	610	0.013	0.911
Field Environment	608	13.767	0.01 *
Population:Field Environment	606	22.893	0.001 **
*Proctolaelaps subcorticalis* Berlese			
Population	610	11.052	0.001 **
Field Environment	608	9.115	0.03 *
Population: Field Environment	606	5.352	0.135
*Tarsonemus endophloeus* Lindq.			
Population	610	3.407	0.02 *
Field Environment	608	4.588	0.03 *
Population: Field Environment	606	0.001	0.784

Field environments are as follows: Logan Canyon, Utah = LC; Locket Meadow, Arizona = LM; Centennial Forest, Arizona = CF. LC is the native environment for Utah populations and LM is the native environment for Arizona populations. CF represents a novel warming environment. ** = Highly significant (*p* < 0.01); * = Significant (*p* < 0.05). These values also corroborate the global community estimates as shown in Table S1.

## Supplementary Materials

The following are available online at https://www.mdpi.com/2075-4450/12/2/131/s1: Figure S1: Total number of successful galleries (i.e., parent beetle pairs) per population for each field site, Figure S2: Graphic showing morphology of mites and their attachment locations on mountain pine beetles. Images Table S1: Mite species list, functional roles, average abundances (per beetle) ± standard error between populations and across field environments, and estimated total number of mites per beetle gallery for each species.
