# Supplementary material for: Field Translocation of Mountain Pine Beetles Suggests Phoretic Mite Communities Are Locally Adapted, and Mite Populations Respond Variably to Climate Warming
Source: Insects. 2021 Feb 2;12(2):131. doi: 10.3390/insects12020131 (PMC7913132; doi:10.3390/insects12020131)
Supplement: Supplementary file 1 [file insects-12-00131-s001.pdf]

Supplementary Material: Field Translocation of Mountain Pine Beetles Suggests Phoretic Mite Communities Are Locally Adapted, and Mite Populations Respond Variably to Climate Warming

Sneha Vissa, David N. Soderberg and Richard W. Hofstetter

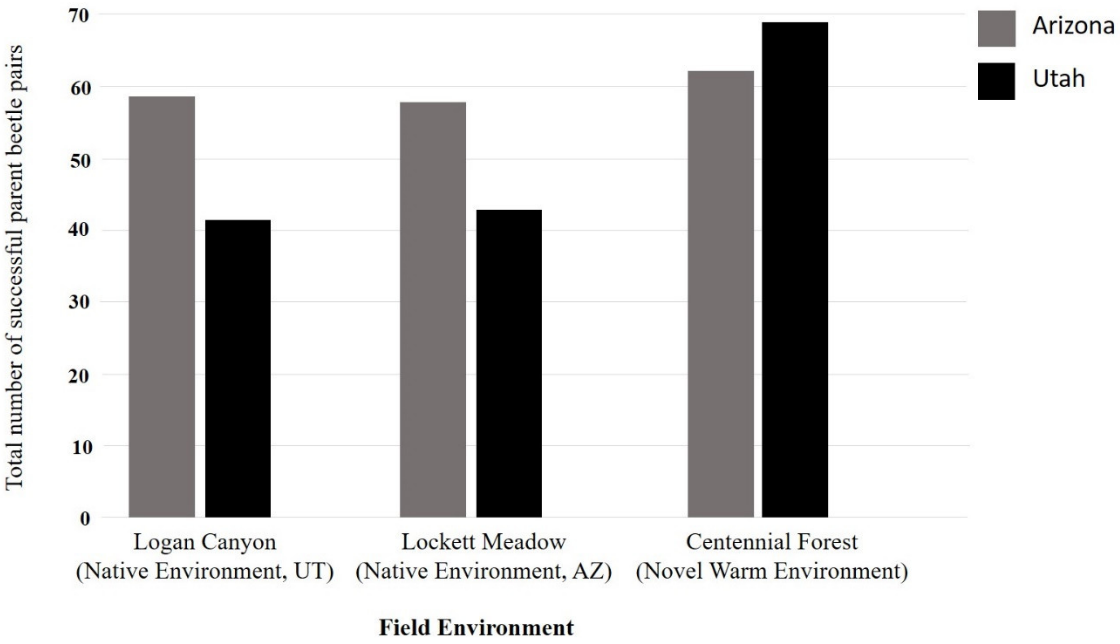

Figure S1. Total number of successful galleries (i.e. parent beetle pairs) per population for each field site; used to estimate number of progeny per parent beetle pair shown in Figure 3; and estimated number of mites per beetle gallery as shown in Table 2 and Table S1.

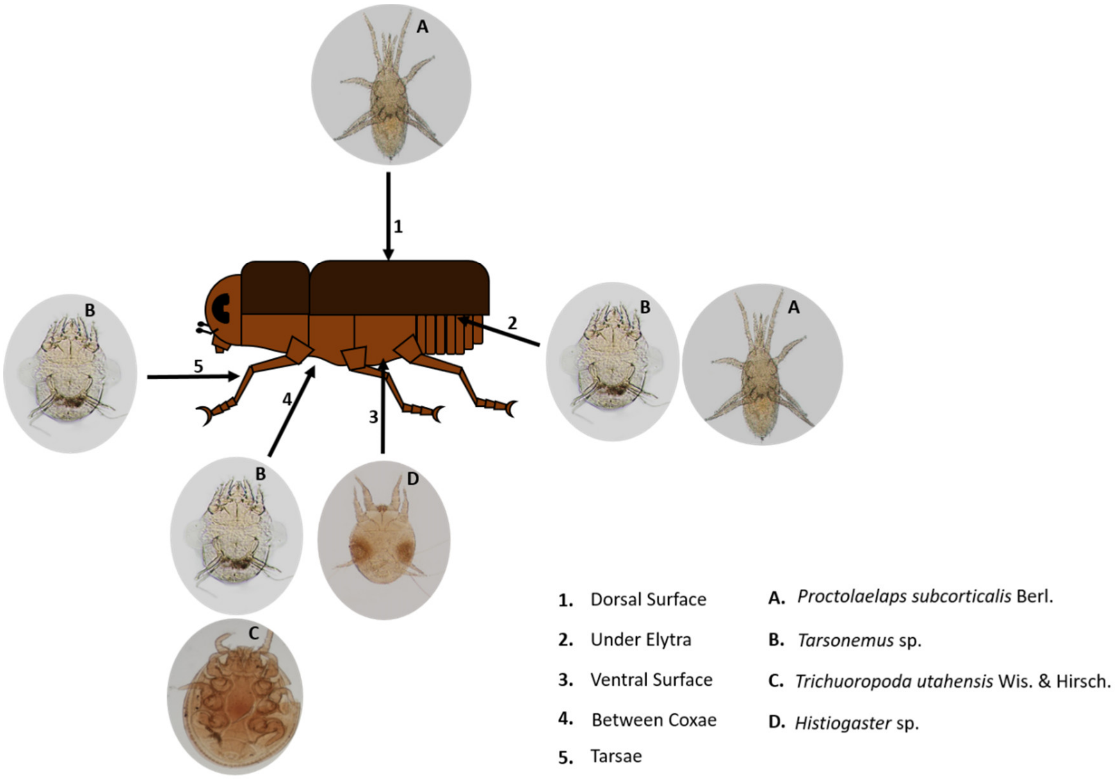

Figure S2. Graphic showing morphology of mites and their attachment locations on mountain pine beetles. Images provided by Sneha Vissa, School of Forestry. Images were taken at the Museum of Northern Arizona imaging lab facilities.

Table S1. Mite species list, functional roles, and average abundances (per beetle) ± standard error between populations and across field environments.

Taxa		Mites Per Beetles and Total Mites Per Beetle Gallery			Mites Per Beetles and Total Mites Per Beetle Gallery		
		AZ			UT		
**Species**	**Mite functional role**	**Native (LM)**	**Translocated (LC)**	**Novel Warm (CF)**	**Native (LC)**	**Translocated (LM)**	**Novel Warm (CF)**
Tarsonemus ips	Fungivore	1.13 (±0.19) **34**	0.85 (±0.16) **21**	2.90 (±0.5) **96**	4.8 (±0.63) **139**	4.77 (±0.75) **110**	2.1 (±0.33) **65**
Tarsonemus endophloeus	Fungivore				0.07 (±0.04) **2**		0.01 (±0.01) **0–1**
Trichouropoda utahensis	Omnivore	0.71 (±0.17) **21**	0.94 (±0.17) **24**	0.91 (±0.2) **30**	0.31 (±0.08) **9**	1.86 (±0.34) **43**	0.46 (±0.09) **14.26**
Proctolaelaps subcorticalis	Predator	0.02 (±0.01) **0–1**	0.11 (±0.03) **3**	0.14 (±0.04) **5**	0.05 (±0.04) **2**		
Histiogaster sp.	Detritivore	0.06 (±0.03) **2**			0.05 (±0.03) **1**	0.03 (±0.01) **0-1**	0.05 (±0.02) **2**

Bolded numbers show an estimated total number of exiting (phoretic) mites associated with each successful beetle gallery for each population within field treatments (based on average no. of mites/beetle and beetle reproductive success) at each field environment rounded to the nearest whole number. See Figure S1 for number of successful galleries per site.
